# Function of Epirubicin-Conjugated Polymeric Micelles in Sonodynamic Therapy

**DOI:** 10.3389/fphar.2019.00546

**Published:** 2019-05-21

**Authors:** Kazuhisa Takemae, Jun Okamoto, Yuki Horise, Ken Masamune, Yoshihiro Muragaki

**Affiliations:** ^1^Institute of Advanced Biomedical Engineering and Science, Tokyo Women’s Medical University, Tokyo, Japan; ^2^Pharmaceutical Division, Kowa Company, Ltd., Tokyo, Japan

**Keywords:** sonodynamic therapy, high-intensity focused ultrasound, reactive oxygen species, triggered high-intensity focused ultrasound, drug-conjugated polymeric micelles

## Abstract

The combinatory use of high-intensity focused ultrasound (HIFU) and epirubicin (EPI)-conjugated polymeric micellar nanoparticles (NC-6300) is thought to be a less invasive and more efficient method of cancer therapy. To investigate the mechanism underlying the combination effect, we examined the effect of trigger-pulsed HIFU (TP-HIFU) and NC-6300 from the perspective of reactive oxygen species (ROS) generation, which is considered the primary function of sonodynamic therapy (SDT), and changes in drug characteristics. TP-HIFU is an effective sequence for generating hydroxyl radicals to kill cancer cells. EPI was susceptible to degradation by TP-HIFU through the production of hydroxyl radicals. In contrast, EPI degradation of NC-6300 was suppressed by the hydrophilic shell of the micelles. NC-6300 also exhibited a sonosensitizer function, which promoted the generation of superoxide anions by TP-HIFU irradiation. The amount of ROS produced by TP-HIFU reached a level that caused structural changes to the cellular membrane. In conclusion, drug-conjugated micellar nanoparticles are more desirable for SDT because of accelerated ROS production and drug protection from ROS. Furthermore, a combination of NC-6300 and TP-HIFU is useful for minimally invasive cancer therapy with cooperative effects of HIFU-derived features, antitumor activity of EPI, and increased ROS generation to cause damage to cancer cells.

## Introduction

The application of ultrasound has greatly improved disease diagnosis and therapeutics in clinical practice. High-intensity focused ultrasound (HIFU) is an important method that causes a large temperature increase at the focal region, causing thermal ablation of tissue (Dubinsky et al., 2008). HIFU is clinically employed for managing prostate cancer, hepatocellular carcinoma, uterine leiomyomas, and breast tumors (Copelan et al., 2015). Although ablation itself has large effects, the higher energy is associated with the risk of adverse effects like skin burns and the possibility of energy absorption in the muscles and bones in the body (Copelan et al., 2015).

Sonodynamic therapy (SDT) is a combination treatment of ultrasound irradiation and sonosensitizer administration that shows potential as a minimally invasive treatment for cancer (Lafond et al., 2018). The sonosensitizer originated from the photosensitizer used for photodynamic therapy and displayed similar functions when combined with ultrasound instead of light (Umemura et al., 1993; Tachibana et al., 2008). Several sonosensitizers have been reported, including the porphyrin complex, rose Bengal, titanium oxide, gold nanoparticles, and anticancer drugs like anthracyclines (Costley et al., 2015).

Although the mechanism of SDT is unclear, it is probably based on the activation of a sonosensitizer via acoustic cavitation and generation of reactive oxygen species (ROS) (Riesz et al., 1985). In photodynamic therapy, the trigger for singlet oxygen generation is the light-based excitation of the photo-sensitizer. In contrast, SDT is thought to be more complex because of the non-direct reaction between the sonosensitizer and ultrasound. Some intermediate steps are related to acoustic cavitation between the initiation of ultrasound irradiation and final ROS generation. Acoustic cavitation involves gas bubbles in the medium generated via ultrasound irradiation. Inertial cavitation refers to the rapid growth and collapse of bubbles, and stable cavitation refers to the sustained oscillatory motion of bubbles (Mitragotri, 2005). A previous study suggested two major mechanisms of ROS generation via acoustic cavitation, referred to as “sono-luminescence” and “pyrolysis” (Misik and Riesz, 2000; Kuroki et al., 2007).

Several ROS are reportedly involved in SDT. Hydroxyl radicals are well-known ultrasound-related ROS (Misik and Riesz, 2000). The superoxide anion is generated from doxorubicin, a widely used anticancer agent (Yumita et al., 1989). Singlet oxygen is generated via SDT and photodynamic therapy (Canavese et al., 2018).

Numerous challenges exist regarding nanoparticle preparation via sonosensitizers (Serpe et al., 2012). Some sono-sensitizers are highly hydrophobic and show a disadvantageous distribution in the body. To overcome these limitations, an approach for forming nanoparticles, which are useful as drug delivery systems and include liposomes, polymers, and poly(lactic-co-glycolic acid), were reported including microbubble encapsulation (McEwan et al., 2014; Yan et al., 2016; Feng et al., 2018; Wang et al., 2018). In addition to improving the distribution and sono-chemical activity, the existence of a particle in a liquid promotes the formation of a cavitation bubble because of its surface roughness (Serpe et al., 2012). As another related technology, liposome preparations that release temperature-sensitive drugs following HIFU irradiation are being examined (Boissenot et al., 2016).

NC-6300 is an EPI-conjugated polyethylene glycol polyaspartate block copolymer. The conjugates form a micellar structure in aqueous media and exhibit the unique feature of pH-dependent drug release. Approximately 80% of EPI was found to be released within 1 h at pH 3 (Harada et al., 2011). This drug delivery system carrier has been confirmed to be safe based on the results of a phase 1 clinical trial (Mukai et al., 2017).

Trigger-pulsed HIFU (TP-HIFU) is a programmed sequence consisting of two ultrasound waves. One is a high-intensity short duration pulse and the other is moderate-intensity long sustaining wave (Yoshizawa et al., 2016; Iwasaki et al., 2018). TP-HIFU irradiation accelerates the generation of microbubble clouds and thermal coagulation (Umemura et al., 2013).

We found that the combinatory use of NC-6300 and TP-HIFU irradiation was much more effective than each single therapy (Maeda et al., 2017). We also conducted clinical safety tests for TP-HIFU and NC-6300 in pet dogs and confirmed the superior safety of this method.

Despite this progress in practical application, the mechanism of the combination effect and functional significance of the drug-polymer conjugate is unclear. In this study, we examined the effect of the combination of TP-HIFU and NC-6300 from the perspective of ROS generation and changes in drug characteristics.

## Materials and Methods

### Reagents

NC-6300 was obtained from NanoCarrier Co., Ltd. (with material transfer agreement) and diluted in Dulbecco’s phosphate-buffered saline (DPBS) to prepare a stock solution (EPI concentration of 2 mM). EPI, distilled water, superoxide dismutase, RPMI 1640 medium, and accutase were from Nacalai Tesque (Kyoto, Japan). DPBS, iron(II) FeSO_4_, H_2_O_2_, xanthine, and xanthine oxidase were from Fujifilm Wako Pure Chemical (Osaka, Japan). Fetal bovine serum was from Gibco (Grand Island, NY, United States). Penicillin-streptomycin solution was from Sigma (St. Louis, MO, United States). Hydroxy phenyl fluorescein (HPF) was from Goryo Chemical (Sapporo, Japan), and Cell Counting Kit-8 (CCK-8) and WST-1 were from Dojindo Laboratories (Kumamoto, Japan). The Annexin V fluorescein isothiocyanate (FITC)-propidium iodide (PI) kit was from Medical and Biological Laboratories (Nagoya, Japan).

### Cell Lines

Human pancreas adenocarcinoma (BxPC-3) cells were obtained from the American Type Culture Collection (Manassas, VA, United States) and human peripheral blood promyeloblast (HL-60) cells were from the Japanese Collection of Research Bioresources Cell Bank. The cells were cultured in RPMI-1640 medium containing 10% fetal bovine serum and penicillin-streptomycin at 37°C and 5% CO_2_ in an incubator.

### HIFU Irradiation

**FIGURE 1:**
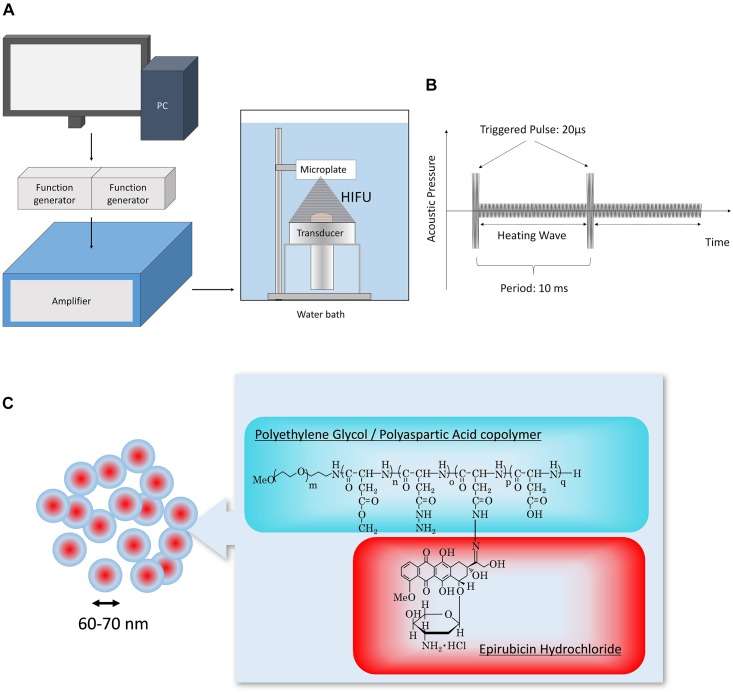
**(A)** Schematic of the experimental setup. HIFU condition (trigger sequence and duration of irradiation) was controlled using original software. The signal generated from the function generator was amplified and then output to the transducer. **(B)** Sequence of TP-HIFU. High-intensity triggering pulse and heating wave were irradiated repeatedly. **(C)** Structure of NC-6300.

The *in vitro* HIFU system was constructed as shown in Figure 1. The transducer was designed using a lead zirconate titanate ceramic element (resonant frequency 1.09 MHz, focus distance from transducer 75 mm) (Abe et al., 2014; Yoshizawa et al., 2016; Maeda et al., 2017). The input signal was amplified with an amplifier (A300, Electronics and Innovation, Rochester, NY, United States) through the transducer, followed by the generation of the sequence by the function generator (WF1974, NF Corporation, Yokohama, Japan). The HIFU conditions (trigger sequence and duration of irradiation) were controlled with original software. The ultrasound intensity (spatial peak temporal average intensity; I_SPTA_, W/cm^2^) was measured using a needle-type hydrophone (HPM05, Precision Acoustics, Dorset, United Kingdom). TP-HIFU is a programmed wave mixture of a short-duration high-intensity triggering pulse (2000 W/cm^2^, 0.02 ms) followed by a heating wave (10–1000 W/cm^2^). N-HIFU was single heating wave irradiation (100 W/cm^2^) without the triggering pulse. The output energy of TP-HIFU at an ultrasound intensity of 100 W/cm^2^ was 10 W.

All irradiation experiments were conducted in a water bath, which was filled with degassed circulating water (DEGASi High Flow, Biotec, Onsala, Sweden). The water temperature and dissolved oxygen concentration were maintained at 25 ± 2°C and <3 mg/mL, respectively (Figure 1).

The sample container for HIFU irradiation was changed as was necessary. Because plastic containers are easily deformed by high temperatures and long-duration irradiation, glass test tubes were used. For short-duration irradiation, 48- and 96-well plates were used to process multiple samples in one experiment.

### Experiment on Degradation Stability and Release of EPI

The drug was dissolved in DPBS and irradiated with HIFU. Next, the drug concentration was measured using high-performance liquid chromatography as previously described for NC-6300 (Harada et al., 2011), with some modifications required for the laboratory conditions.

### Cytotoxicity Test

BxPC-3 cells were treated with accutase and detached from the plate at 37°C for 15 min. The detached cells were collected using centrifugation and diluted to 1 × 10^5^ cells/mL. An aliquot of the sample (0.34 mL) was added to each well of the 96-well plate. The HIFU irradiation group was placed in the water bath and HIFU irradiation was performed. During the experiment, the temperature of the water bath was maintained at 25 ± 2°C. HIFU-irradiated samples were dispensed at 0.1 mL into a 96-well microplate and cultured for the specified duration at 37°C and 5% CO_2_ in an incubator. After cell culture, 10 μL of CCK-8 was added and incubated for an additional 4 h at 37°C. The cell number was measured using a microplate reader (Infinite M1000 PRO, Tecan, Männedorf, Switzerland) at a wavelength of 438 nm. Cell viability was calculated using untreated cells as a control.

### Hydroxyl Radical Detection Assay

To detect hydroxyl radicals, fluorescence was measured using HPF. HPF was added to the sample solution at a final concentration of 0.025 mM. The sample solution was irradiated with HIFU after dispersion into a 48-well microplate (1.3 mL). The sample was dispensed into a 96-well plate for fluorescence intensity measurement. The fluorescence of the decomposed substance (HPF to fluorescein, ex. 490 nm, em. 515 nm) was measured in a microplate-reader (Infinite M1000 PRO). Three samples were included in each group, and the average value and standard deviation of the fluorescence changes were calculated.

### Super Oxide Detection Assay

To measure superoxide anions, the WST-1 method was used (Ukeda et al., 1999). WST-1 was added to DPBS at a final concentration of 0.2 mM, and then NC-6300 or EPI was added to the 48-well microplate (1.3 mL) and TP-HIFU irradiation was performed (100 W/cm^2^, 0.5–3 min) at 25°C. The absorbance at a wavelength of 438 nm was measured using a UV-VIS-near infrared spectrophotometer (V-670DS, JASCO, Tokyo, Japan). Three samples were evaluated for each group, and the average value and standard deviation of absorbance changes were calculated before and after HIFU irradiation.

### Flow Cytometer Analysis

The cellular accumulation of annexin V-FITC and PI was monitored using flow cytometry to detect changes in the membrane structure caused by ROS. HL-60 cells were cultured in RPMI-1640 medium containing 10% fetal bovine serum and penicillin-streptomycin, and diluted to 1 × 10^5^ cells/mL, and hydroxyl radicals or superoxide anions were generated by the Fenton reaction or xanthine/xanthine oxidase reaction. In the Fenton reaction, H_2_O_2_ solution and FeSO_4_ solution were added to a final concentration of 0.005–0.02 mM. In the xanthine/xanthine oxidase reaction, xanthine and xanthine oxidase were added to final concentrations of 0.005–0.02 mM and 40 mU/mL, respectively. The reaction was carried out at 37°C and 5% CO_2_ in an incubator. After incubation for 6 h, annexin-FITC and PI were added according to the manufacturer’s instructions and the luminescence of FITC and PI was monitored with a flow cytometer (LSR Fortessa X-20, BD Biosciences, Franklin Lakes, NJ, United States) and analyzed using FlowJo v10 software (FlowJo LLC, Ashland, OR, United States).

Statistical analysis was carried out by using JMP software ver. 13.0.0 (SAS Institute, Cary, NC, United States).

## Results

We evaluated the effect of TP-HIFU irradiation and compared the amounts of hydroxyl radicals generated by N-HIFU and TP-HIFU. TP-HIFU generated more hydroxyl radicals even with short-duration irradiation compared to N-HIFU. Hydroxyl radical levels also increased as the HIFU intensity increased.

The cytoreductive effect of TP-HIFU was examined using BxPC-3 cells. The cells were irradiated with TP-HIFU or N-HIFU after detachment using accutase treatment. After 48 h of incubation, the survival of adherent cells significantly differed between the TP-HIFU and N-HIFU treatment groups.

**FIGURE 2:**
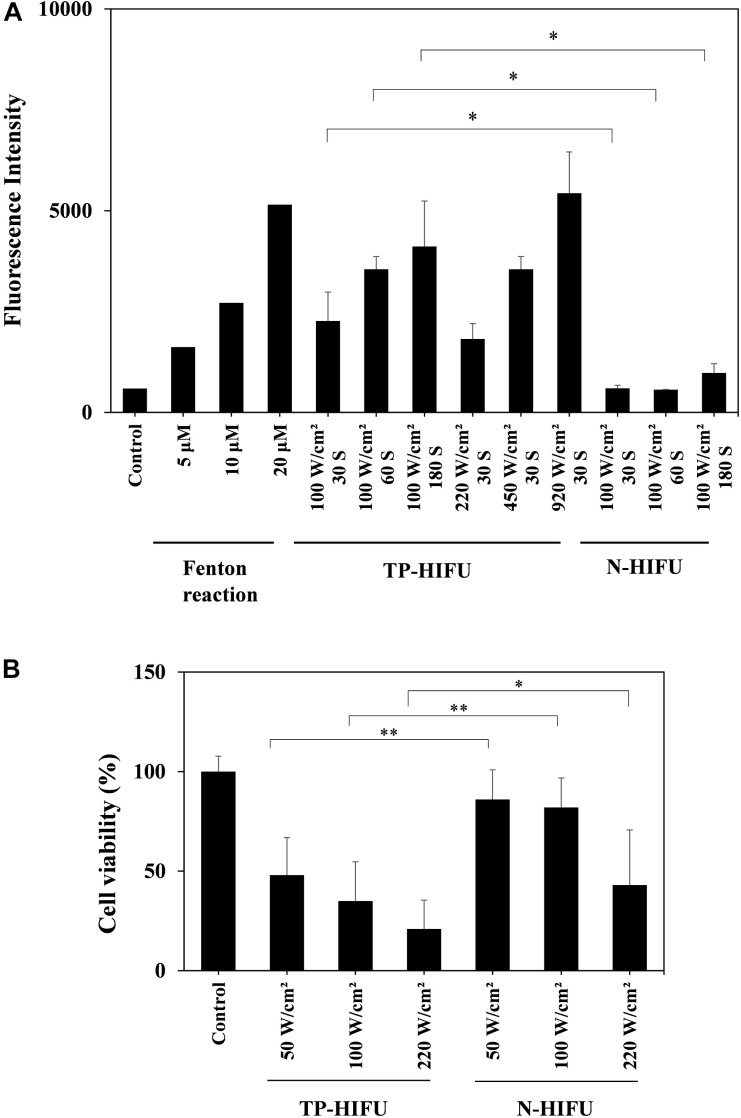
**(A)** Hydroxyl radical measurement by HPF method. In the Fenton reaction sample, H_2_O_2_ solution and FeSO_4_ solution were both added to HPF solution to final concentration of 5, 10, and 20 μM, respectively. The fluorescence intensity of the fluorescein released from HPF by hydroxyl radicals was measured with a microplate reader (ex./em. = 490/515 nm). HIFU irradiation groups were collected for five or more irradiation samples and the results are presented as the mean ± standard deviation. ^∗^*P* < 0.05 non-parametric comparison for each pair by Wilcoxon test. **(B)** Cell viability assay of BxPC-3 cell line. Suspension of detached BxPC-3 cells were dispensed into a 96-well microplate for HIFU irradiation. Viable-cell counting was carried out by CCK-8 method. Each group was collected for 20 or more irradiation samples and the results are presented as the mean ± standard deviation. ^∗^*P* < 0.05, ^∗∗^*P* < 0.01, non-parametric comparison for each pair by Wilcoxon test.

Based on these results, TP-HIFU is a useful method for generating cavitation and cell killing effects after short-duration irradiation (Figure 2).

To confirm the stability of EPI against TP-HIFU, the change in the concentration of EPI upon TP-HIFU irradiation was measured. EPI showed slight decrease in concentration by TP-HIFU irradiation. However, EPI degradation was suppressed when NC-6300 was irradiated.

**FIGURE 3:**
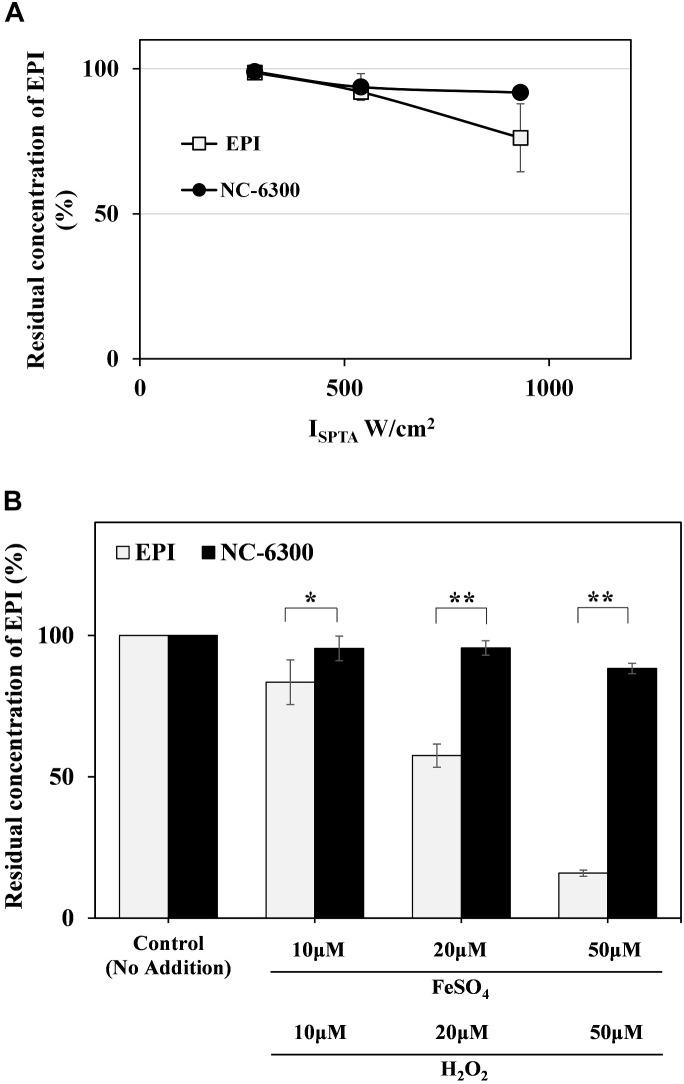
**(A)** EPI degradation test by TP-HIFU irradiation. The sample solution containing NC-6300 or EPI (0.1 mg/mL EPI concentration) was irradiated 10 min by TP-HIFU in test tube made of glass. Residual amount of EPI was calculated with non-irradiated sample set to 100%. The irradiation groups were collected as three samples and presented as the mean ± standard deviation. **(B)** EPI degradation test by Fenton reaction. The sample solution containing NC-6300 or EPI (0.01 mM EPI concentration) was treated with Fenton reaction in test tube. The residual amount of EPI was measured by HPLC (*n* = 2–3) and presented as the mean ± standard deviation. ^∗^*P* < 0.05, ^∗∗^*P* < 0.01, comparison for each pair by Tukey–Kramer test.

Because hydroxyl radicals may be related to EPI degradation upon HIFU irradiation, we examined the change in the concentration of EPI by hydroxyl radical addition using the Fenton reaction. The Fenton reaction is a source of hydroxyl radicals produced by the mixing of iron ion and H_2_O_2_. The EPI concentration was decreased by the generation of hydroxyl radicals. However, NC-6300 was relatively stable after the generation of hydroxyl radicals (Figure 3).

To investigate the potency of NC-6300 as a sonosensitizer, we examined the detection of superoxide anion after HIFU irradiation. Because we could not detect superoxide anion using the electron spin resonance method, we used the spectroscopic method as previously reported (Ukeda et al., 1999).

**FIGURE 4:**
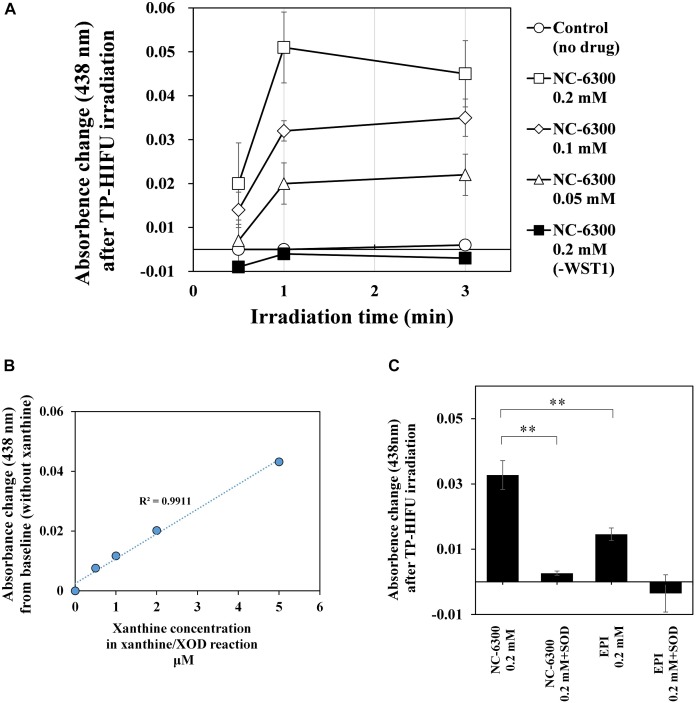
Measurement of the amount of superoxide anions by UV spectrophotometer. The sample solution containing 0.2 mM WST-1 and the indicated concentration of NC-6300 or EPI (as EPI concentration) was irradiated by TP-HIFU. **(A)** TP-HIFU irradiation to NC-6300 in various concentration and at different irradiation times. **(B)** Correlation with xanthine concentration in xanthine/xanthine oxidase reaction and absorbance change at 438 nm. **(C)** Comparison of the generation of superoxide anion with NC-6300 and EPI. Superoxide dismutase (SOD) was added to confirm generation of the superoxide anion. The absorbance change was measured at 438 nm and that of non-irradiated sample was subtracted. The irradiation groups were collected as three samples and presented as the mean ± standard deviation. ^∗∗^*P* < 0.01, comparison for each pair by Tukey–Kramer test.

We measured the amount of superoxide anion generated by TP-HIFU irradiation using a UV-VIS-near infrared spectrophotometer. As a result, a concentration-dependent increase in superoxide anion was observed when NC-6300 was irradiated by TP-HIFU. The TP-HIFU irradiation to NC-6300 without WST-1, the superoxide indicator reagent, showed no absorbance change. The amount of superoxide reached a maximum level within 1 min of irradiation under this experimental condition. Moreover, N-6300 showed a superior ability to generate superoxide anion than did EPI. Addition of superoxide dismutase suppressed the absorbance change after TP-HIFU irradiation. The generated amount of superoxide anion was estimated to be in the μM range compared with the concentration of xanthine in the xanthine/xanthine oxidase reaction (Figure 4).

**FIGURE 5:**
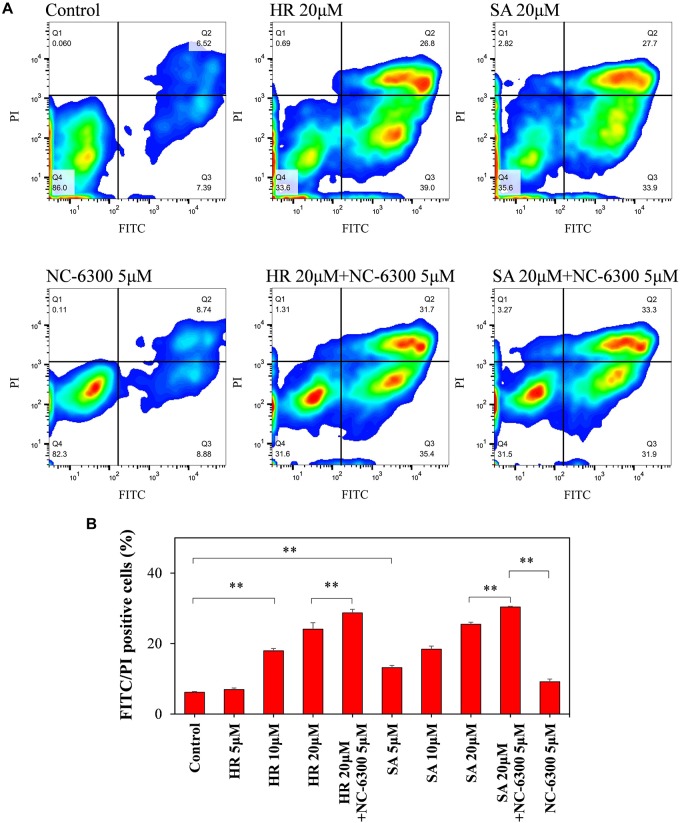
Flow cytometry analysis of HL-60 cells. The cell suspension was dispensed into test tubes and hydroxyl radicals (HR) or superoxide anions (SA) were added by Fenton reaction or xanthine/xanthine oxidase reaction. After 6-h incubation at 37°C, the samples were measured by flow cytometry. **(A)** FITC and PI intensity changes were monitored to confirm changes in the cell membrane upon extracellular addition of ROS and NC-6300. **(B)** Percentage of the number of FITC- and PI-double-positive cells (Q2 region). Three samples were collected form each group and the results are presented as the mean ± standard deviation. ^∗∗^*P* < 0.01, comparison for each pair by Tukey–Kramer test.

To investigate the extracellular function of ROS at the estimated level after TP-HIFU irradiation, we monitored the apoptosis-related changes in the cells using flow cytometry. HL-60 cells were incubated following addition of hydroxyl radicals or superoxide anions using the Fenton reaction or xanthine/xanthine oxidase reaction with or without NC-6300. After 6 h of incubation, FITC- and PI-positive cells were increased after adding both hydroxyl radicals and superoxide anions. This change was accelerated by adding NC-6300 (5 μM), which was not observed after adding NC-6300 alone (Figure 5).

## Discussion

To investigate the mechanism of the antitumor effects of NC-6300 and TP-HIFU demonstrated previously (Maeda et al., 2017), we evaluated the function of TP-HIFU. We found that hydroxyl radical generation was promoted by TP-HIFU irradiation (Figure 2A). We assume that the increase in cavitation bubbles by TP-HIFU increases the production of hydroxyl radicals after sonolysis (Misik and Riesz, 2000). It has been reported that 8–15 μM of hydroxyl radicals were generated by sonolysis under the irradiation conditions of 30 W, 1,650 kHz, and 30 and 60 s (Nakamura et al., 2011). In contrast, the output energy of TP-HIFU (Figure 2A) was one-third (10 W) that in the aforementioned study and the same level of hydroxyl radicals were obtained. The amount of hydroxyl radicals increased with increasing irradiation intensity, even with short-duration irradiation, suggesting that higher hydroxyl radical generation occurs when the HIFU intensity is increased. These results indicate that TP-HIFU irradiation produces more hydroxyl radicals in a short duration and the amount of ROS will be >20 μM under clinical conditions.

Additionally, higher cytoreductivity was observed with the application of TR-HIFU irradiation (Figure 2B). Although the triggering pulse in TP-HIFU is 2000 kw/cm^2^, the duration is very short (0.02 ms/cycle, duty 0.2%). Thus, the total output energy is nearly the same as that of N-HIFU which produces only a heating wave. In the preliminary experiment, the temperature of the microplate well was not increased beyond 35°C throughout irradiation and was within the non-thermal level. Despite these conditions, the difference between TP-HIFU and N-HIFU was clearly noticeable. To clarify the involvement of hydroxyl radicals in cytotoxicity, we carried out an additional experiment with 1 W (10 W/cm^2^) of TP-HIFU irradiation and a hydroxyl radical scavenger. We confirmed cytotoxicity at 1 W, and the effect was suppressed after addition of 2 mM mannitol or histidine (Supplementary Figure S1).

**FIGURE 6:**
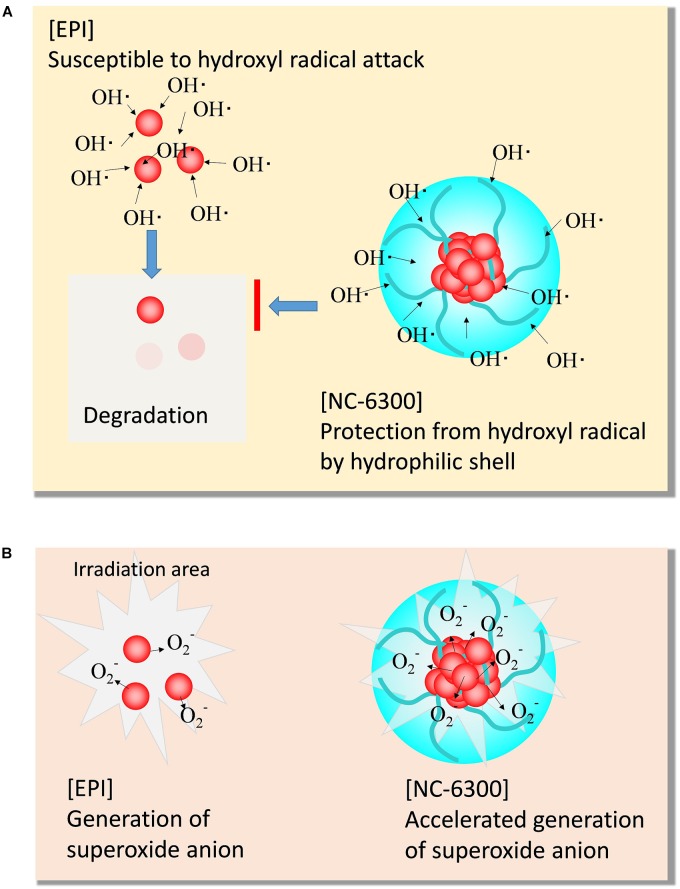
Scheme of the characteristic function of NC-6300 in SDT. **(A)** Protective effect from hydroxyl radical. **(B)** Promoting effect of superoxide anion.

The results of EPI degradation by strong TP-HIFU irradiation and similar results obtained from Fenton reaction suggest that EPI degradation by TP-HIFU occurred mainly because of hydroxyl radicals. In contrast, NC-6300 suppressed the degradation of EPI (Figure 3). This may be because EPI localization at the inner core and surrounding hydrophilic moiety created by the PEG-polyamino acid polymer blocked hydroxyl radical attack EPI (Figure 6). It is unclear whether other drugs are protected in a similar manner, and further experiments are necessary.

EPI was not released from NC-6300 even at high-power irradiation (data not shown). The results indicate that the hydrazone bond which conjugates the EPI and polymer was not cleaved by TP-HIFU irradiation even at a high energy. No significant change was observed in the particle size in the nanoparticles after HIFU irradiation (data not shown). These results indicate that cavitation generated under the current conditions cannot cleave the conjugates.

In contrast, the generation of superoxide anions increased following addition of NC-6300 (Figure 4). Although EPI itself functioned as a sonosensitizer for generating superoxide anion, NC-6300 generated more superoxide anion than EPI. We assumed that EPI localized in the inner core at high concentration acted promptly in the ROS generation process (Figure 6). However, the fundamental mechanism underlying the generation of superoxide radicals from epirubicin is unclear and warrants further study (Figure 6).

Reactive oxygen species function is thought to be the key component of SDT. In this study, we estimated the levels of hydroxyl radicals and superoxide anions at the laboratory scale. Hydroxyl radicals are thought to reach up to 20 μM and superoxide anions are estimated to be present at up to one tenth of the hydroxyl radicals. The results of flow cytometry suggested that the cells were susceptible to ROS and that apoptosis-like changes occurred at the outer membrane at 20 μM of hydroxyl radicals and super oxide anions. The hypothesis that ROS might attack from the extracellular matrix is one of the possible causes of this combination treatment. NC-6300 is moved from blood vessel to tumor region by EPR effect and may be first accumulated in the extracellular matrix (Chida et al., 2018). We assume that some intervention of ROS could occur from extracellular matrix to membrane (Supplementary Figure S3). Further studies are necessary to determine the following mechanism after the ROS have attacked from outside of the cellular membrane.

Furthermore, intracellular ROS levels were not determined in this study. Temporal increases in intracellular ROS levels were observed after 30 min of ultrasound irradiation to metal-porphyrin complexes (Giuntini et al., 2018), despite the short (<1 s) lifetime of ROS. Combined use of folate-polyethylene-glycol decorated gold nanoparticles and ultrasound irradiation shows remarkable cytotoxicity and ROS production in folate receptor overexpressing cells. However, the effect was suppressed when the cell uptake of the particles was blocked by co-incubation with excess free folate (Brazzale et al., 2016). It is also reported that ROS generated by SDT is related to autophagy (Li et al., 2017; Kou et al., 2017). These findings indicates the possibility of a late-phase effect of ROS after TP-HIFU irradiation, warranting further experiments.

In addition, we assume that more therapeutic effects are expected in this therapy because we confirmed cooperative HIFU-derived features (ablation, mechanical destruction, and cavitation), an anticancer drug, and ROS within the dose that is clinically safe. First, cell destruction by TP-HIFU was immediately observed (Supplementary Figure S2, 24 h). Second, the anticancer drug suppressed cell proliferation to inhibit tumor growth. Finally, induced apoptosis further decreased the cancer cell population (Supplementary Figure S2, 72 h). One possible clinical benefit is that the anticancer drug is no longer necessary for cell reduction because HIFU takes over this function and reduces the dose of drug required from the levels used for cell killing to that for suppressing cell “growth”. Thus, the safety margin for drug administration should be reevaluated. Chemo-radio therapy is the most widely used therapy. However, the mechanism of radiotherapy does not provide an immediate effect for physical reduction (such as in surgery and thermal coagulation).

One of the benefits of using drug delivery system carrier conjugation with anticancer drugs is improved safety, which leads to improved quality of life. However, additional non-clinical data are required for each target organ to determine their suitability for treatment with nano-micelles. In some cases, the drug alone be the best treatment. TP-HIFU irradiation may enhance the accumulation of micelles in cancer tissues, which requires further analysis. Moreover, a diagnostic technology that can precisely detect smaller target cancer colonies is required.

It has been reported that various types of cancer-associated cells are localized in the tumor microenvironment (Junttila and De Sauvage, 2013). It is thought that there is a specific condition for each cancer-associated cell according to its differentiation and localization. For example, metastatic cells which are about to separate from the primary tumor mass and migrate to another location might form small aggregates or single cells. In contrast, cancer-associated fibroblasts may be in the form of clumps and attached to the tumor (Hwang et al., 2008), suggesting that ablation is the most efficient form of irradiation. To cover such various microenvironments, combinations of different effects such as the treatment described in this study are thought to be useful (Supplementary Figure S4).

In conclusion, drug-conjugated nano-micelles are more desirable for SDT because of accelerated ROS production and drug protection from ROS. Combinatorial treatment with NC-6300 and TP-HIFU is a useful approach for minimally invasive cancer therapy, combining the cooperative effects of HIFU-derived features, anticancer activity of EPI, and increased ROS generation to damage cancer cells.

## Author Contributions

KT, JO, YH, KM, and YM conceived and designed the study. KT performed the experiments. KT and YM organized and wrote the manuscript.

## Conflict of Interest Statement

KT was employed at Kowa Company, Ltd., Tokyo, Japan. The remaining authors declare that the research was conducted in the absence of any commercial or financial relationships that could be construed as a potential conflict of interest.

## References

[B1] Abe N. Nakamoto H. Suzuki T. Muragaki Y. Iseki H. (2014). Ex vivo evaluation of high-intensity focused ultrasound with ultrasonic-induced cavitation bubbles. *J. Med. Ultrason.* 41 3–9. 10.1007/s10396-013-0469-9 27277627

[B2] Boissenot T. Bordat A. Fattal E. Tsapis N. (2016). Ultrasound-triggered drug delivery for cancer treatment using drug delivery systems: from theoretical considerations to practical applications. *J. Control. Release* 241 144–163. 10.1016/j.jconrel.2016.09.026 27667179

[B3] Brazzale C. Canaparo R. Racca L. Foglietta F. Durando G. Fantozzi R. (2016). Enhanced selective sonosensitizing efficacy of ultrasound-based anticancer treatment by targeted gold nanoparticles. *Nanomedicine* 11 3053–3070. 10.2217/nnm-2016-0293 27627904

[B4] Canavese G. Ancona A. Racca L. Canta M. Dumontel B. Barbaresco F. (2018). Nanoparticle-assisted ultrasound: a special focus on sonodynamic therapy against cancer. *Chem. Eng. J.* 340 155–172. 10.1016/j.cej.2018.01.060 30881202 PMC6420022

[B5] Chida T. Miura Y. Cabral H. Nomoto T. Kataoka K. Nishiyama N. (2018). Epirubicin-loaded polymeric micelles effectively treat axillary lymph nodes metastasis of breast cancer through selective accumulation and pH-triggered drug release. *J. Control. Release* 292 130–140. 10.1016/j.jconrel.2018.10.035 30391405

[B6] Copelan A. Hartman J. Chehab M. Venkatesan A. M. (2015). High-intensity focused ultrasound: current status for image-guided therapy. *Semin. Intervent. Radiol.* 32 398–415. 10.1055/s-0035-1564793 26622104 PMC4640913

[B7] Costley D. Mc Ewan C. Fowley C. McHale A. P. Atchison J. Nomikou N. (2015). Treating cancer with sonodynamic therapy: a review. *Int. J. Hyperthermia* 31 107–117. 10.3109/02656736.2014.992484 25582025

[B8] Dubinsky T. J. Cuevas C. Dighe M. K. Kolokythas O. Hwang J. H. (2008). High-intensity focused ultrasound: current potential and oncologic applications. *Am. J. Roentgenol.* 190 191–199. 10.2214/ajr.07.2671 18094311

[B9] Feng Q. Li Y. Yang X. Zhang W. Hao Y. Zhang H. (2018). Hypoxia-specific therapeutic agents delivery nanotheranostics: a sequential strategy for ultrasound mediated on-demand tritherapies and imaging of cancer. *J. Control. Release* 275 192–200. 10.1016/j.jconrel.2018.02.011 29474964

[B10] Giuntini F. Foglietta F. Marucco A. M. Troia A. Dezhkunov N. V. Pozzoli A. (2018). Insight into ultrasound-mediated reactive oxygen species generation by various metal-porphyrin complexes. *Free Radic. Biol. Med.* 121 190–201. 10.1016/j.freeradbiomed.2018.05.002 29738830

[B11] Harada M. Bobe I. Saito H. Shibata N. Tanaka R. Hayashi T. (2011). Improved anti-tumor activity of stabilized anthracycline polymeric micelle formulation, NC-6300. *Cancer Sci.* 102 192–199. 10.1111/j.1349-7006.2010.01745.x 21040218

[B12] Hwang R. F. Moore T. Arumugam T. Ramachandran V. Amos K. D. Rivera A. (2008). Cancer-associated stroma fibroblasts promote pancreatic tumor progression. *Cancer Res.* 68 918–926. 10.1158/0008-5472.can-07-5714 18245495 PMC2519173

[B13] Iwasaki R. Nagaoka R. Yoshizawa S. Umemura S. (2018). Selective detection of cavitation bubbles by triplet pulse sequence in high-intensity focused ultrasound treatment. *Jpn. J. Appl. Phys.* 57 1–6. 10.1158/0008-5472.can-07-5714 18245495 PMC2519173

[B14] Junttila M. R. De Sauvage F. J. (2013). Influence of tumour micro-environment heterogeneity on therapeutic response. *Nature* 501 346–354. 10.1038/nature12626 24048067

[B15] Kou J. Y. Y. Li Y. Zhong Z. Y. Y. Jiang Y. Q. Q. Li X. S. S. Han X. B. B. (2017). Berberine-sonodynamic therapy induces autophagy and lipid unloading in macrophage. *Cell Death Dis.* 8:e2558. 10.1038/cddis.2016.354 28102849 PMC5386349

[B16] Kuroki M. Hachimine K. Abe H. Shibaguchi H. Maekawa S. Yanagisawa J. (2007). Sonodynamic therapy of cancer using novel sonosensitizers. *Anticancer Res.* 27 3673–3677. 17970027

[B17] Lafond M. Yoshizawa S. Umemura S. (2018). Sonodynamic therapy: advances and challenges in clinical translation. *J. Ultrasound Med.* 38 567–580. 10.1002/jum.14733 30338863

[B18] Li Q. Kang J. Xiong X. Cao W. Liu Y. Li Y. (2017). Protoporphyrin IX-mediated sonodynamic therapy promotes autophagy in vascular smooth muscle cells. *Oncol. Lett.* 14 2097–2102. 10.3892/ol.2017.6394 28789437 PMC5530015

[B19] Maeda M. Muragaki Y. Okamoto J. Yoshizawa S. Abe N. Nakamoto H. (2017). Sonodynamic therapy based on combined use of low dose administration of epirubicin-incorporating Drug delivery system and focused ultrasound. *Ultrasound Med. Biol.* 43 2295–2301. 10.1016/j.ultrasmedbio.2017.06.003 28705555

[B20] McEwan C. Fowley C. Nomikou N. McCaughan B. McHale A. P. Callan J. F. (2014). Polymeric microbubbles as delivery vehicles for sensitizers in sonodynamic therapy. *Langmuir* 30 14926–14930. 10.1021/la503929c 25409533

[B21] Misik V. Riesz P. (2000). Free radical intermediates in sonodynamic therapy. *Ann. N. Y. Acad. Sci.* 899 335–348. 10.1111/j.1749-6632.2000.tb06198.x 10863551

[B22] Mitragotri S. (2005). Innovation - Healing sound: the use of ultrasound in drug delivery and other therapeutic applications. *Nat. Rev. Drug Discov.* 4 255–260. 10.1038/nrd1662 15738980

[B23] Mukai H. Kogawa T. Matsubara N. Naito Y. Sasaki M. Hosono A. (2017). A first-in-human Phase 1 study of epirubicin-conjugated polymer micelles (K-912/NC-6300) in patients with advanced or recurrent solid tumors. *Invest. New Drugs* 35 307–314. 10.1007/s10637-016-0422-z 28054329

[B24] Nakamura K. Ishiyama K. Ikai H. Kanno T. Sasaki K. Niwano Y. (2011). Reevaluation of analytical methods for photogenerated singlet oxygen. *J. Clin. Biochem. Nutr.* 49 87–95. 10.3164/jcbn.10-125 21980223 PMC3171684

[B25] Riesz P. Berdahl D. Christman C. L. (1985). Free radical generation by ultrasound in aqueous and nonaqueous solutions. *Environ. Health Perspect.* 64 233–252. 10.1289/ehp.8564233 3007091 PMC1568618

[B26] Serpe L. Foglietta F. Canaparo R. (2012). Nanosonotechnology: the next challenge in cancer sonodynamic therapy. *Nanotechnol. Rev.* 1:10.

[B27] Tachibana K. Feril L. B. Ikeda-Dantsuji Y. (2008). Sonodynamic therapy. *Ultrasonics* 48 253–259. 10.1016/j.ultras.2008.02.003 18433819

[B28] Ukeda H. Kawana D. Maeda S. Sawamura M. (1999). Spectrophotometric assay for superoxide dismutase based on the reduction of highly water-soluble tetrazolium salts by xanthine-xanthine oxidase. *Biosci. Biotechnol. Biochem.* 63 485–488. 10.1271/bbb.63.485 27393255

[B29] Umemura S. Yoshida S. Takagi R. Inaba Y. Yasuda J. (2013). Enhancement of focused ultrasound treatment by acoustically generated microbubbles. *Jpn. J. Appl. Phys.* 52 1–6. 10.7150/thno.11848 26379791 PMC4568453

[B30] Umemura S. Yumita N. Nishigaki R. (1993). Enhancement of ultrasonically induced cell damage by a gallium-porphyrin complex, ATX-70. *Jpn. J. Cancer Res.* 84 582–588. 10.1111/j.1349-7006.1993.tb00179.x 8320175 PMC5919169

[B31] Wang X. Yan F. Liu X. Wang P. Shao S. Sun Y. (2018). Enhanced drug delivery using sonoactivatable liposomes with membrane-embedded porphyrins. *J. Control. Release* 286 358–368. 10.1016/j.jconrel.2018.07.048 30075210

[B32] Yan S. Lu M. Ding X. Chen F. He X. Xu C. (2016). HematoPorphyrin monomethyl ether polymer contrast agent for ultrasound/photoacoustic dual-modality imaging-guided synergistic high intensity focused ultrasound (HIFU) therapy. *Sci. Rep.* 6 31833–31833. 10.1038/srep31833 27535093 PMC4989155

[B33] Yoshizawa S. Matsuura K. Takagi R. Yamamoto M. Umemura S. (2016). Detection of tissue coagulation by decorrelation of ultrasonic echo signals in cavitation-enhanced high-intensity focused ultrasound treatment. *J. Ther. Ultrasound* 4 1–13. 10.1186/s40349-016-0060-0 27081486 PMC4831115

[B34] Yumita N. Nishigaki R. Umemura K. Umemura S. (1989). Increase in the generation of superoxide radicals and in the inhibitory effect on yoshida sarcoma of anthracycline antitumor agents by ultrasound. *Nihon Gan Chiryo Gakkai Shi* 24 63–68. 2544650

