# Supplementary material for: Function of Epirubicin-Conjugated Polymeric Micelles in Sonodynamic Therapy
Source: Front Pharmacol. 2019 May 21;10:546. doi: 10.3389/fphar.2019.00546 (PMC6536629; doi:10.3389/fphar.2019.00546)
Supplement: Supplementary file 1 [file Data_Sheet_1.pdf]

Supplementary Material

Function of Epirubicin-Conjugated Polymeric Micelles in Sonodynamic Therapy

Kazuhisa Takemae[1,2], Jun Okamoto[1], Yuki Horise[1], Ken Masamune[1], Yoshihiro Muragaki[1,*]

[1]Institute of Advanced Biomedical Engineering and Science, Tokyo Women's Medical University, Tokyo, Japan

[2]Pharmaceutical Division, Kowa Company, Ltd., Tokyo, Japan

***Correspondence:**
Dr. Yoshihiro Muragaki
ymuragaki@twmu.ac.jp

1.1 Supplementary Figures

(A)

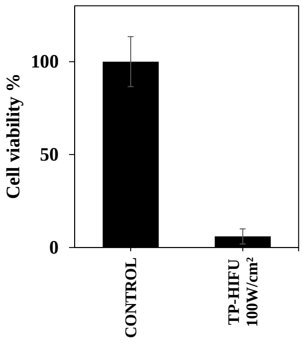

(B) No scavenger Mannitol Histidine

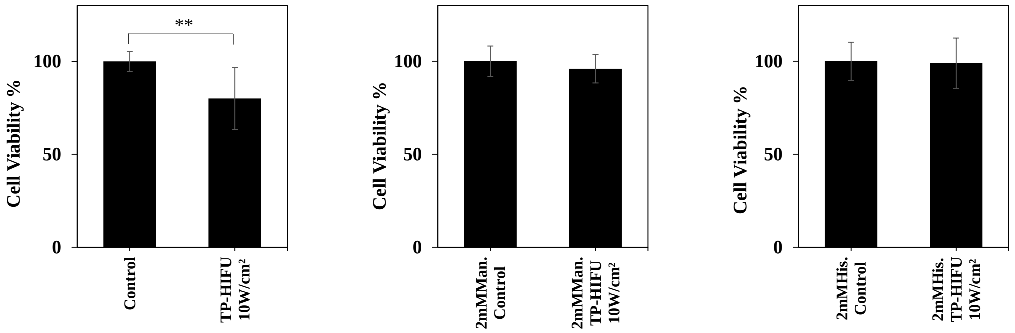

Supplementary Figure S1. Cell viability assay of HL-60 cell line.

(A) The HL-60 cells were diluted to 1× 10^5 cells/mL. An aliquot of the sample (0.34 mL) was added to each well of the 96-well plate. The HIFU irradiation group was placed in the water bath and HIFU irradiation was performed (100W/cm^2 (10W), 30sec). (B) The HL-60 cells were diluted to 2 × 10^5 cells/mL. An aliquot of the sample (0.34 mL) was added to each well of the 96-well plate. The HIFU irradiation group was placed in the water bath and HIFU irradiation was performed (10W/cm^2 (1W), 180sec). Man: Mannitol, His: Histidine.

HIFU-irradiated samples were dispensed at 0.1 mL into a 96-well microplate and 10 μL of CCK-8 was added and incubated for an additional 4 h at 37°C. The cell number was measured using a microplate reader (Infinite M1000 PRO, Tecan, Männedorf, Switzerland) at a wavelength of 438 nm, and cell viability was calculated using untreated cells as a control. Each group was collected for 10 irradiation samples and the results are presented as the mean ± standard deviation. ** $P<0.01$, non-parametric comparison for each pair by Student's t-test.

A 24hr incubation B 72hr incubation

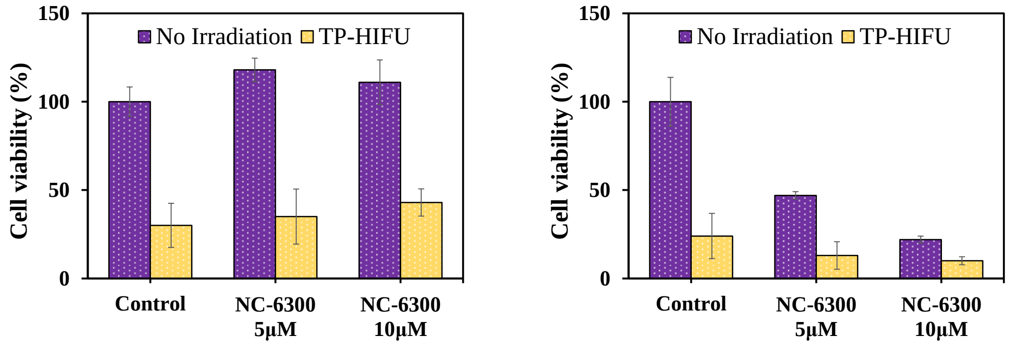

Supplementary Figure S2. Cell viability assay of BxPC-3 cell line.

BxPC-3 cells were treated with accutase and then detached from the plate at 37°C for 15 min. The detached cells were collected by centrifugation and suspended to the medium. After NC-6300 was added, the suspension was diluted to 1×10^5 cells/mL. An aliquot of the sample (0.34 mL) was added to each well of the 96-well plate. The HIFU irradiation group was placed in the water bath and HIFU irradiation was performed. During the experiment, temperature of the water bath was maintained at 37 ± 2°C. HIFU-irradiated samples were dispensed at 0.1 mL into a 96-well microplate and cultured for the specified duration (24, 72hr) at 37°C and 5% CO_2 in an incubator. After cell culture, 10 μL of CCK-8 was added and incubated for an additional 4 h at 37°C. The cell number was measured using a microplate reader and cell viability was calculated using untreated cells as a control. Each group was collected 9-10 samples and presented as the mean ± standard deviation.

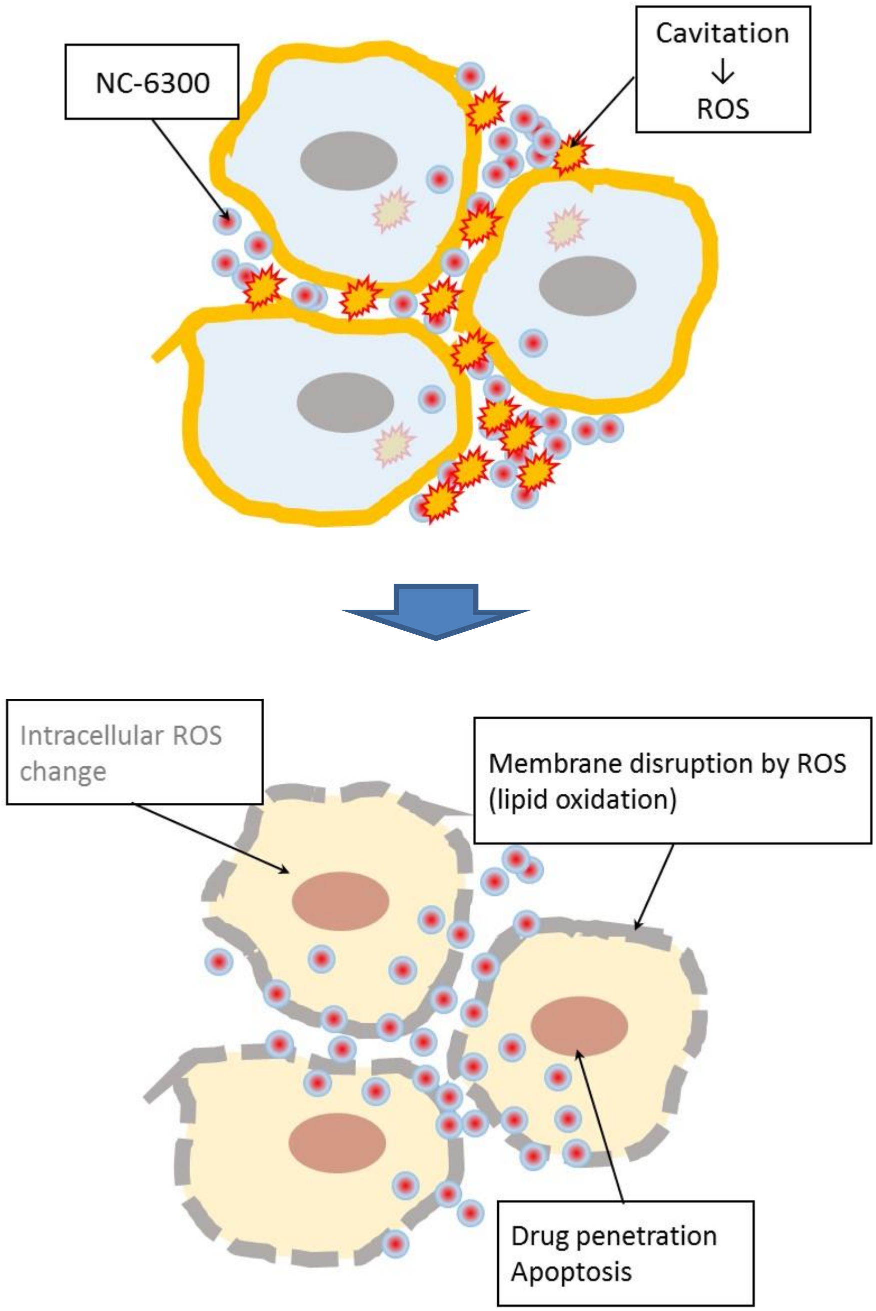

Supplementary Figure S3. Schematic of the hypothetical mechanism of extracellular ROS function.

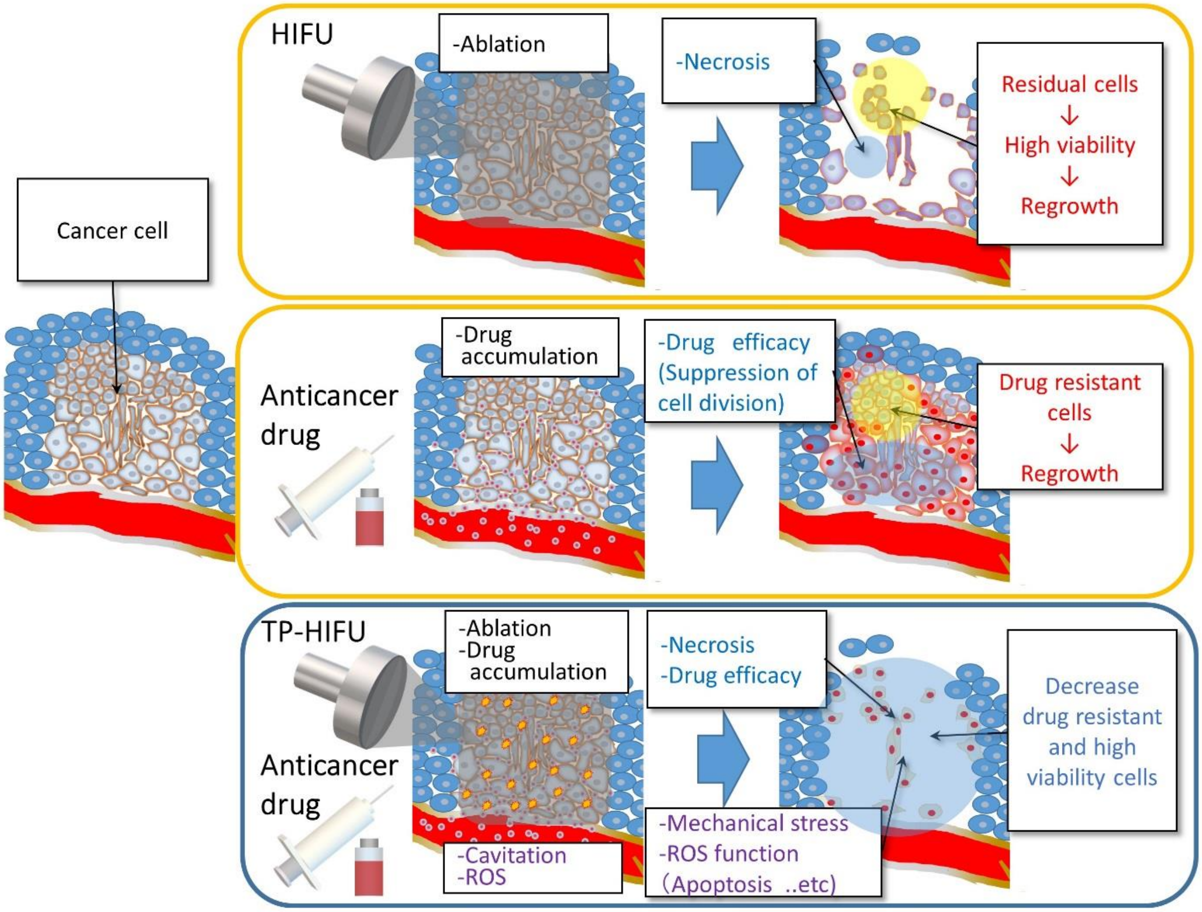

Supplementary Figure S4. Schematic of the hypothetical mechanism of TP-HIFU and NC-6300 combination treatment.
